# BSA-Coated Gold Nanorods for NIR-II Photothermal Therapy

**DOI:** 10.1186/s11671-021-03627-7

**Published:** 2021-11-29

**Authors:** Shubi Zhao, Yiqun Luo, Zong Chang, Chenchen Liu, Tong Li, Lu Gan, Yong Huang, Qinchao Sun

**Affiliations:** 1 National Center for International Research of Bio-Targeting Theranostics, Guangxi Key Laboratory of Bio-Targeting Theranostics, Collaborative InnovationCenter for Targeting Tumor Diagnosis and Therapy, Guangxi Medical University, Nanning, 530021 Guangxi China; 2 Research Laboratory for Biomedical Optics and Molecular Imaging, Shenzhen Institutes of Advanced Technology, Chinese Academy of Sciences, Shenzhen, 518055 Guangdong China

**Keywords:** BSA-coated gold nanorods, Near-infrared-II, Photothermal therapy

## Abstract

The second near infrared window is considered to be the optimal optical window for medical imaging and therapy as its capability of deep tissue penetration. The preparation of the gold nanorods with long wavelength absorption and low cytotoxicity is still a challenge. A series gold nanorods with large aspect ratio have been synthesized. Strong plasma absorption in the second near infrared window from 1000 to 1300 nm could be observed. The biocompatibility of the synthesized gold nanorods is dramatically improved via coating by bovine serum albumin (BSA), while the optical properties of which remains. The breast cancer tumor-bearing mouse could be well treated by the prepared gold nanorods with the NIR-II light intensity as low as 0.75 W/cm^2^. In summary, these results demonstrate the feasibility of using low illumination dose to treat tumor in the NIR-II region via the large aspect ratio gould nanoparticles.

**Supplementary Information:**

The online version contains supplementary material available at 10.1186/s11671-021-03627-7.

## Introduction

Gold nanoparticles have been attracting wide interest in biomedical researches as the brilliant biocompatability and the low cytotoxicity. For instance, the gold nanoparticles with high X-ray attenuation efficiency were found to be promising for computed tomography (CT)-based tumor diagnosis [1, 2]. Furthermore, gold nanoparticles exhibit excellent optical properties which is known as surface plasmon resonance (SPR) effect. The gold nanoparticles could efficiently convert the photon energy to thermal energy for cancer therapy in the presence of surface plasmon resonance light [3, 4]. Therefore, various gold nanoparticles with adjustable size and morphology have been developed for photothermal ablation of tumors, for instance gold nanorods, gold nanoshells, and gold nanocages [5–7]. In particular, gold nanorods (AuNR) with adjustable anisotropic shape and size have been widely studied since their excellent photothermal stability, biocompatibility, and strong absorption in NIR region [8]. It is well known that near-infrared light could penetrate into biological tissues more effectively than visible light, as the longer the light wavelength, the lower the light scattering loss [9]. In addition, the rod-shaped nanoparticles have been found with dramatically enhanced tumor permeability and longer blood circulation time, resulting higher tumor accumulation [10, 11]. However, the significance drawback for applying gold nanorods to photothermal therapy (PTT) is the high-power laser irradiation, which would induce highly damage of normal tissue (maximum allowable light intensity exposure) [12]. It has been proven that the PTT in the second near infrared window (NIR-II, 1000–1700 nm) has much greater tissue penetration depth than that in NIR-I (700–1000 nm), as the much lower light scattering in NIR-II [13–17]. Therefore, the PTT nano-platform in the NIR-II is expected to achieve more effective PTT treatment of tumor and to have great clinical application potential for more complex tumor therapy. However, the preparation of the gold nanorods with long wavelength absorption and low cytotoxicity is still a great challenge. Here, we report the synthesis of gold nanorods by the seedless method with absorption peaks in the second window of near-infrared window (1000–1300 nm). Surface modification was introduced via coating with BSA to reduce the cytotoxicity. The aspect ratio of prepared gold nanorods (AuNR@BSA) was characterized by transmission electron microscope (TEM) and dynamic light scattering (DLS). The breast cancer tumor-bearing mouse model was used to test the photothermal therapeutic effect of AuNR@BSA. We found that the tumor could be well treated with light intensity as low as 0.75 w/cm^2^.

## Materials and Methods

### Materials

Gold chloride trihydrate (HAuCl_4_·3H_2_O) (99.9%), Hexadecyl trimethyl ammonium bromide (CTAB) (99%), Nitric acid (GR, 65–68%), and Hydrogen peroxide solution (GR, 30%) were received from Shanghai Aladdin biological technology Co. Ltd. Sodium borohydride (NaBH_4_) (97%) and silver nitrate (AgNO_3_) (99.8%) were received from Shanghai Lingfeng Chemical Reagent Co. Ltd. Hydrochloric acid (HCl) (38%) was received from Dongguan Dongjiang Chemical Reagent Co. Ltd. Hydroquinone (99%) was received from Energy Chemical. Bovine Serum Albumin (98%) was obtained from Sigma-Aldrich. Sodium hydroxide (AR,96%) was received from Greagent.

RPMI 1640 Medium and Penicillin–Streptomycin were purchased from HyClone. Phosphate-buffered solution (PBS) was purchased from Corning. Pancreatin was purchased from Coolaber. Foetal bovine serum (FBS) was purchased from Gibco. 4T1 cells were provided by Research Center for Biomedical Optics and Molecular Imaging in the Shenzhen Institute of Advanced Technology, Chinese Academy of Science. Dojindo Chemical Technology (Shanghai) Co., Ltd supplied Cell Counting Kit-8 (CCK-8) for Cell proliferation and toxicity test. The Millipore ultrapure water was used throughout the experiment.

### Preparation of AuNR@CTAB Nanoparticles

The synthesis of gold nanorods is conducted as follows: 0.4 mL of HAuCl_4_(aq) (10 mM) and 10 mL of CTAB(aq) (0.1 M) were added to 23–33 µL of AgNO_3_(aq) (100 mM). Then, 10–30 µL of HCl (1.2 M) and 525 µL of aqueous hydroquinone (0.1 M) were added to the growth solution under gentle mixing. The color of the growth solution turned from orange to a very light yellow. After 15 min of stirring, 10–40 µL of freshly prepared ice-cold NaBH_4_(aq) (10 mM) solution was injected into the growth solution. The mixture was stirred for 30 s and aged for 18 h at room temperature. The AuNR@CTAB was then washed by PBS twice.

### Preparation of AuNR@BSA

First, we add a certain amount of CTAB to adjust its concentration in AuNR@CTAB solution to 1 mM, and then ultrasound completely dissolves CTAB. 3 mL AuNR@CTAB is slowly added to 3 mL BSA solution (10 mg/mL), and the mixed solution is sonicated for 30 min. After centrifugation at 9500×r for 40 min, the supernatant was replaced with 6 mL BSA solution (5 mg/mL), and then, the pH was adjusted to 11–12 with sodium hydroxide (2 M), stirred for at least 18 h. After that, the synthesized AuNR@BSA was centrifuged at 9500×r for 40 min, then washed twice with PBS, and dissolved in PBS for further use.

### Characterizations of AuNR@CTAB and AuNR@BSA Nanoparticles

The morphology analysis of gold nanorods was obtained by Beijing Zhongke Baice Co., Ltd. through Talos F200X electron microscope to obtain TEM images. Zetasizer Nano ZS (Malvern, UK) was used to study the size distribution and zeta potential of various nanoparticles by DLS. The UV–Vis absorption spectrum was determined by UV-2700 Ultraviolet–Visible Spectrophotometer (SHIMADZU, Japan).

For the morphology characterization of AuNR@BSA inside tumor, 100 µL of AuNR@BSA (OD = 25 at 1064 nm) was injected into tumor sites with irradiation about 10 min, after that the treated tumors were collected. Non-treated tumor was collected as control. The collected tumors were incubated in 2.5% glutaraldehyde solution (Coolaber.co., Beijing, China) for transmission electron microscopy (Beijing Zhongke Baice Co., Ltd). Fourier transform infrared spectroscopy(FT-IR) pattern and X-ray diffraction (XRD) patterns of AuNR samples were obtained by Beijing Zhongke Baice Co., Ltd.

### Measurement of the Photothermal Performance of AuNR@CTAB and AuNR@BSA

The gold nanorod solution was diluted to different OD at 1064 nm (0.5, 1, 1.5, and 2), and the PBS was used as a blank control. The gold nanorods (500 µL) were irradiated with a 1064 nm laser (Haoliangtech, Shanghai, China) at a power intensity of 0.35–1 W/cm^2^ for 30 min. The temperature was recorded with an infrared thermal imager (FLUKE TI25).

### Photostability of AuNR@BSA

To test the photostability, the absorption spectra of AuNR@BSA were measured as a function of the irradiation time. The AuNR@BSA (OD = 1) were irradiated under NIR laser (1064 nm, 0.5 w/cm^2^). From 0 to 10 min, the spectrum was recorded every minutes. The photothermal cycle test was also carried out as the AuNR@BSA solution (0.5 mL) irradiated with every 10 min laser irradiations on and off (1064 nm, 0.5 W/cm^2^), and the temperature change was recorded.

### Cellular Culture

Murine breast cancer cell line (4T1 cells) was cultured in RPMI 1640 containing 10% FBS and 100 U/mL penicillin or 100 µg/mL streptomycin. The culture environment is 37 °C, and the humidification condition is 5% CO_2_.

### In Vitro* Cytotoxicity Assessment of Gold Nanoparticles*

The CCK-8 assay was used to identify the cytotoxicity of gold nanorods. 4T1 cells were pre-seeded into 96-well plates (5 × 10^3^ per well) and incubated for 24 h. Subsequently, 10 μL of various concentrations of AuNR@CTAB and AuNR@BSA were added and incubated for another 24 h. After washing with PBS twice, 10 μl CCK-8 solution was added to each well and incubated for 40 min, followed by measuring the absorbance at 450 nm with a microplate reader.

For phototoxicity, 4T1 cells were pre-seeded into a 96-well plate (5 × 10^3^ per well) and incubated for 24 h, then the cells were irradiated with NIR laser (1064 nm, 0.75 W/cm^2^, 10 min) and further incubated for 24 h. After that, 10 μL CCK-8 solution was added into each well and incubated for a further 40 min at 37 °C. Then, a microplate reader was used to detect the absorbance of each well at 450 nm.

### Tumor-Bearing Mouse Model

All BALB/c mice were purchased from Beijing Vital River Laboratory Animal Technology Co. Ltd. All animal experiment procedures were carried out following the standard procedures approved by Shenzhen Institute of Advanced Technology Committee of the Chinese Academy of Sciences. The tumor model was established by subcutaneously injecting 4T1 cells (2 × 10^6^) into the back of mice. Animal studies were conducted when the tumor volume reaches approximately 100 mm^3^.

### In Vivo* Blood Circulation and Biodistribution*

For circulation time measurement, first, 200 μL of AuNR@BSA was intravenously injected into the tail vein of BALB/c mice, and then 20 μL of blood was collected at 0.25, 2, 4, 6, 8, 12, 36, and 48 h, and diluted with 30 μL PBS to obtain 50 μL blood sample. About 400 μL concentrated HNO_3_ (chromatographic grade) was added, the lid was tighten and digested at 90 °C for 2 h. After cooling to room temperature, 150 μL H_2_O_2_ (chromatographic grade) was slowly dropped in, and then heated to 90 °C for 1 h without cover. In the end, the solution was diluted to 5 mL by ultrapure water. The concentration of Au ion was measured by Inductively Coupled Plasma Optical Emission Spectroscopy (ICP-OES) after passing through 0.44 mm nylon syringe filter.

For biodistribution measure, about 200 μL AuNR@BSA was intravenously injected into the tail vein of BALB/c mice. After 24 h, the mouse was killed and the heart, liver, spleen, lung, and kidney were taken and dried in an oven at 80 °C. Before digestion, each organ was weighed, 800 μL concentrated HNO_3_ (GR) was added, and heated to 90 °C for 2 h. After cooling to room temperature, 200 μL H_2_O_2_ (GR) was slowly added dropwise, heated at 90 °C for 1 h, and then, the solution was diluted to 10 mL by ultrapure water. Finally, concentration of Au ion was measured by ICP-OES after passing through 0.44 mm nylon syringe filter.

### Photothermal Treatment Efficiency

In order to evaluate the thermal therapeutic effect of AuNR@BSA, the tumor-bearing mice with 4T1 tumors were randomly divided into four groups with the tumor volume about 100 mm^3^: (1) AuNR@BSA, (2) AuNR@BSA + Laser; (3) Only Laser (4) Blank control. An infrared thermal imager was used to record the infrared thermal image of the tumor site. The tumor volume and body weight of mice were recorded before and after treatment, respectively. The tumor volume can be calculated according to the normal equation (volume = width^2^ × length/2). Two weeks later, the mice were killed, and the tumors were isolated.

### Data Analysis

SPSS 16.0 statistical software was used for data analysis. The measurement data were expressed as mean ± d, the comparison between groups was done by analysis of variance, and the comparison of count data was performed by Chi-square test. *P* < 0.05 was considered statistically significant.

## Results and Discussion

### Synthesis and Characterization of AuNR@CTAB

**Fig. 1:**
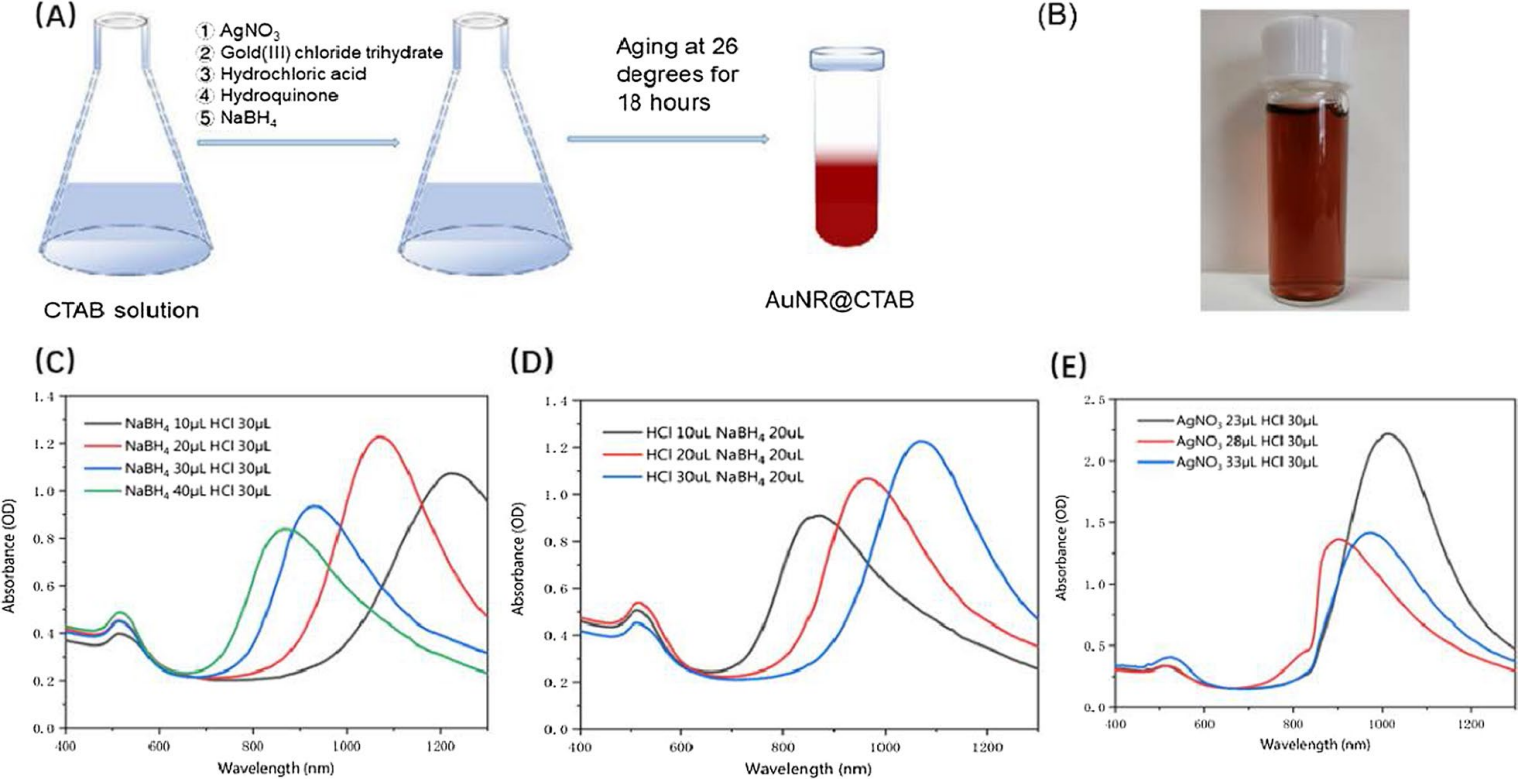
The photoproperties of AuNR@CTAB at different synthesis conditions. **a** The preparation of AuNR@CTAB. **b** The image of CTAB-coated gold nanorods (AuNR@CTAB), **c** UV–vis spectra of prepared AuNR@CTAB with varying NaBH_4_ concentration, **d** UV–vis spectra of prepared AuNR@CTAB with varying HCl concentration **e** UV–vis spectra of prepared AuNR@CTAB with varying AgNO_3_ concentration

It was found that the smaller gold nanorods, the better pharmacokinetics and lower cytotoxicity [18]. However, the SPR absorption peak of gold nanorods is highly related to the aspect ratio, the larger the aspect ratio the lower energy of the SPR peak. In order to synthesize AuNR with large aspect ratio in the meanwhile keep the size as small as possible, the synthesis parameters were optimized such as the surfactant concentration, the pH of the growth solution, and the reducing agent concentration. The NaBH_4_(aq) is a kind of strong reducing agent that forms Au nucleus via LaMer burst nucleation, followed by rapid random attachment of Au ions and intra-particle maturation [19]. As the mole quantity of NaBH_4_ increasing, the maximum absorption peak of gold nanorods undergoes a blue shift from 1223 to 865 nm (Fig. 1C). The pH of the growth solution is also a key parameter to control the growth of gold nanorods, with is adjusted via the amount of hydrochloric acid [20]. The maximum absorption peak of gold nanorods was found to be gradually red-shifted from 871 to 1070 nm while increasing the quantity of hydrochloric acid (Fig. 1D). Furthermore, Ag^+^ is considered to be able to control the growth direction of gold nanorods, and the lower the Ag^+^ concentration the longer absorption wavelength of the SPR peak could be realized (Fig. 1E). In the end, the synthesized gold nanorods are in wine-red, as shown in Fig. 1B. Therefore, considering the synthesis efficiency of gold nanorods and the availability of laser light source, we chose gold nanorods with a maximum absorption peak at 1064 nm for photothermal treatment of tumors.

### Synthesis and Characterization of AuNR@BSA

Cetyltrimethylammonium bromide (CTAB) is the most widely used compound for the synthesis of gold nanorods with precise lengths and aspect ratios. However, CTAB has significant cytotoxicity when the concentration is higher than 1–10 μM. The application of the CTAB-coated gold nanorods (AuNR@CTAB) in biomedicine has been greatly restricted [21]. Furthermore, the colloidal stability of CTAB-coated gold nanorods in aqueous solution is dramatically affected by temperature, which is easy to be crystallized at low temperatures [22]. Given reducing the cytotoxicity of CTAB and improving its stability, several approaches have been proposed to replace CTAB during the gold nanorods synthesis process or to functionalize the CTAB-coated gold nanorods. Using polymers, peptides, surfactants, and lipids to modify nanoparticle surface, most of these strategies use thiolated molecules or electrostatic interaction forces to bind to the gold surface [23]. Proteins are the most promising options as the advantages of colloidal stability, biocompatibility, and further functionalization. [26]

**Fig. 2:**
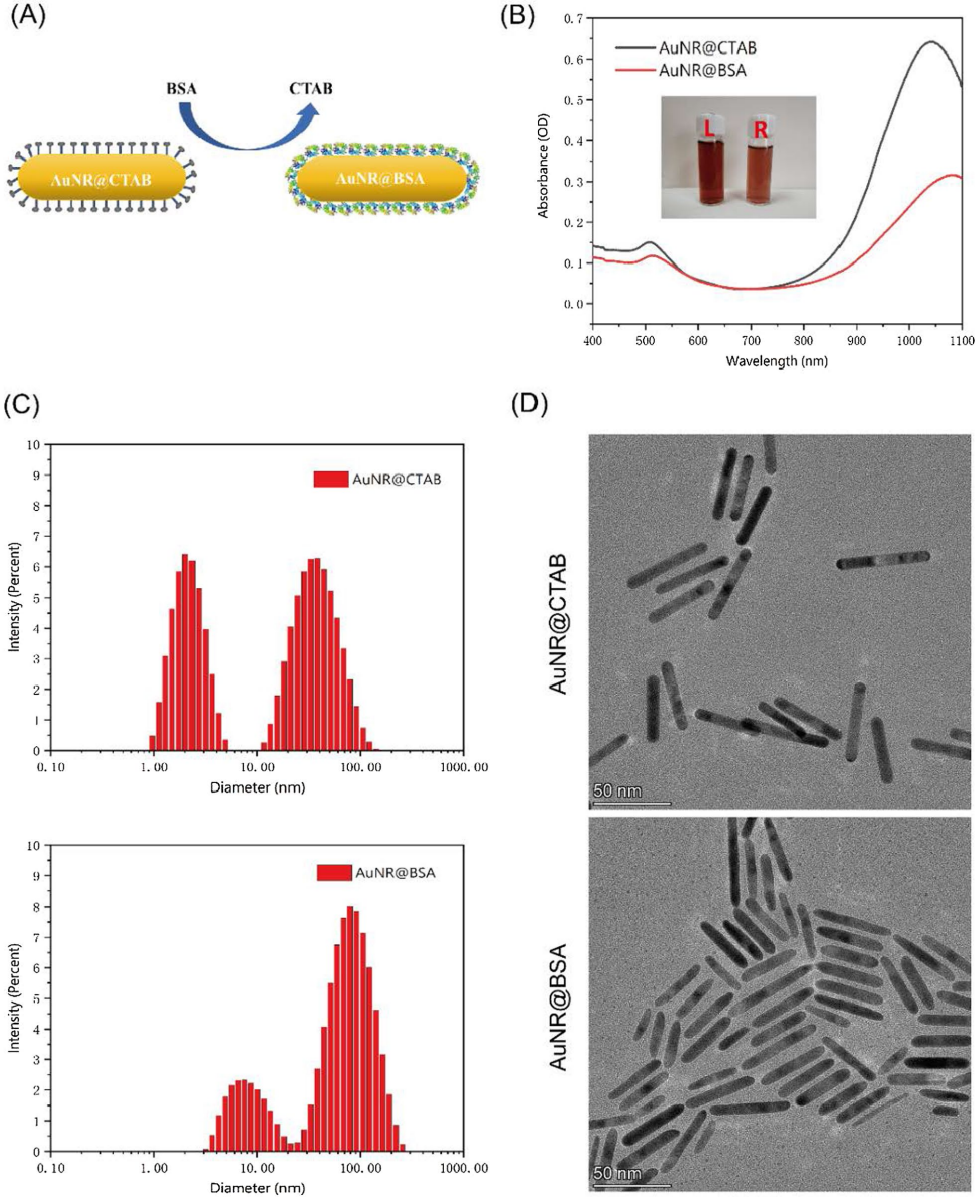
Characterization of AuNR@BSA and AuNR@CTAB. **a** Preparation of AuNR@BSA. **b** UV–Vis spectra of AuNR@CTAB (L) and AuNR@BSA (R). **c** The DLS intensity measurement of AuNR@CTAB and AuNR@BSA. **d** The TEM images of AuNR@CTAB and AuNR@BSA

The CTAB- and BSA-wrapped gold nanorods are depicted in Fig. 2A. The maximum absorption peak of AuNR@BSA is around 1064 nm, which is about 30 nm red-shifted compared to that of AuNR@CTAB (Fig. 2B). The zeta potential of the gold nanorods changed from positive to negative via replacing the CTAB coating to BSA (Additional file 1: Fig. S1). From the FTIR spectra of the AuNR@BSA and AuNR@CTAB (Additional file 1: Fig. S2), we could find that two characteristic peaks of at 1649 cm^−1^ and 1539 cm^−1^ in the case of AuNR@BSA, which were attributed to the amide I and amide II vibrational bands of BSA. The dynamic light scattering (DLS) was also applied to analyze the hydrodynamic size of the AuNR@CTAB and AuNR@BSA; furthermore, the morphology of AuNR@BSA and AuNR@CTAB were characterized by transmission electron microscopy (TEM) as shown in Fig. 2C, D. Two peaks could be clearly found from scattering intensity measurement of DLS, one with hydrodynamic size about 3.10 (± 0.85) nm and the other about 57.45 (± 24.22) nm for AuNR@CTAB. However, in the case of AuNR@BSA, the peaks shift to 8.64 (± 3.80) nm and 89.24 (± 42.24) nm. We could find that the shape of AuNR remains similar after coating with BSA, except the ends becomes slightly rounded (Fig. 2C, D). For in vivo therapy applications, the critical size of nanoparticles is limited to below 100 nm [25]. Beyond this size, the ability of nanoparticles to penetrate tumors will be limited; therefore, the presented gold nanorods would be an ideal tumor therapy candidate [24]. As shown in Additional file 1: Fig. S3, the characteristic peaks of Au can be clearly observed in the XRD pattern, from the (111), (200), (220) and (311) planes of Au nanoparticles.

### In Vitro* Photothermal Effect of Gold Nanorods*

**Fig. 3:**
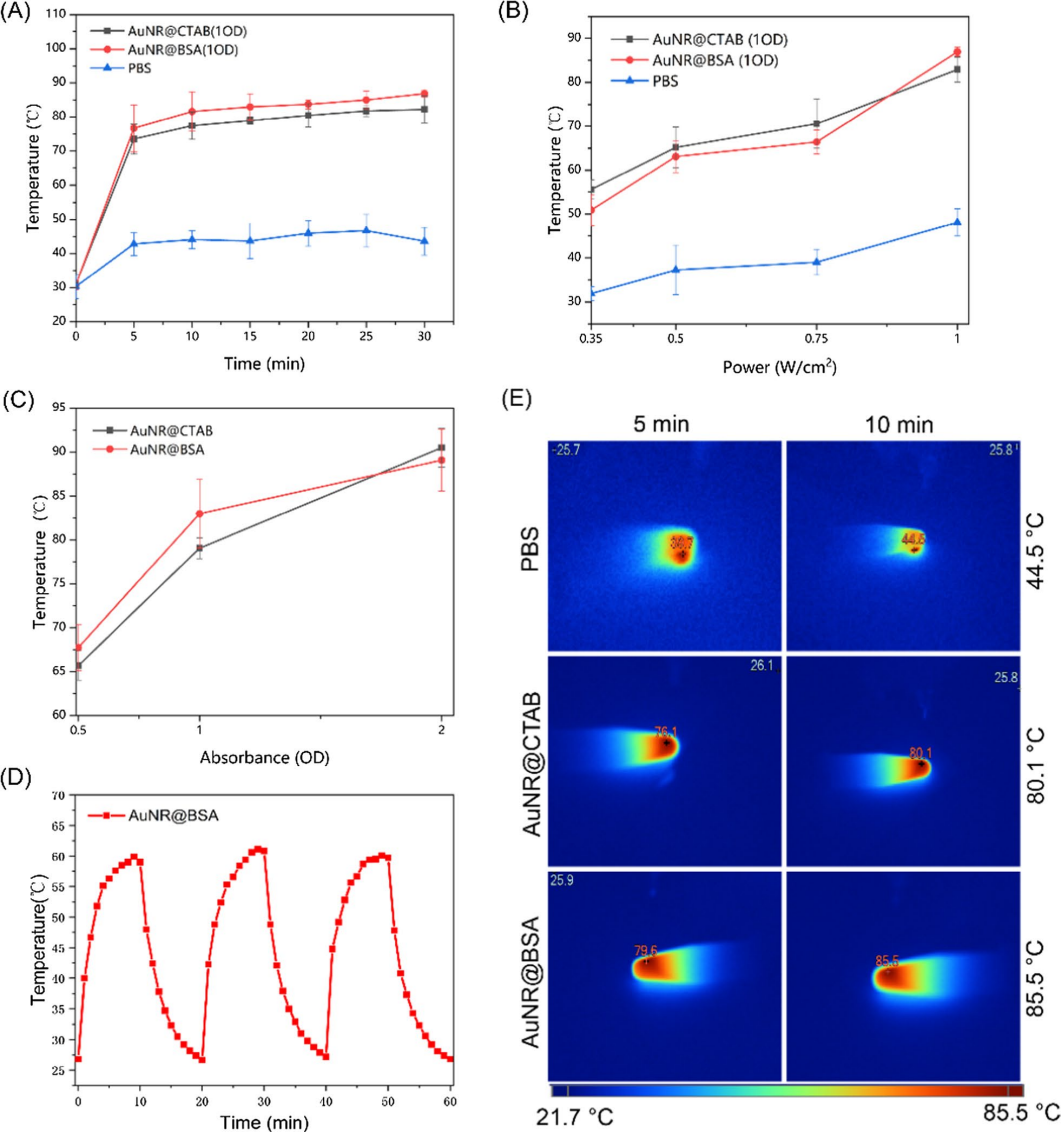
In vitro evaluation of the photothermal effect of AuNR@BSA. **a** The photothermal temperature of AuNR@BSA and AuNR@CTAB as a function of laser irradiation time (SPR absorbance about 1, 1064 nm, 1 W/cm^2^). **b** NIR-triggered temperature increasement of PBS, AuNR@CTAB (OD = 1), and AuNR@BSA (OD = 1) as a function of laser irradiation intensity (1064 nm, 0.35 to 1 W/cm^2^). **c** NIR-triggered temperature increasement of AuNR@BSA and AuNR@CTAB with different absorbance for 10 min laser irradiation (1064 nm, 1 W/cm^2^). **d** Photothermal conversion of AuNR@BSA (OD = 1) under three cycles of irradiation (1064 nm, 0.5 W/cm^2^). **e** Thermal image of PBS, AuNR@CTAB (OD = 1), AuNR@BSA (OD = 1) under irradiated with NIR laser (1064 nm, 1 W/cm^2^) at a time interval of 5 and 10 min. (mean ± SD, n = 3)

The 1064 nm diode laser as the most economic NIR-II light source is considered to be the optimal wavelength for photothermal therapy. Therefore, the ideal NIR-II gold nanoplatform for efficient photothermal therapy is considered to be characterized as strong SPR absorption at 1064 nm, high photothermal efficiency, and excellent photothermal stability. To investigate the photothermal effect of the prepared AuNR@BSA, PBS and different OD (= 0.5, 1, 1.5, 2) of AuNR@BSA were excited at 1064 nm with light intensity from 0.35 to 1 W/cm^2^ for 30 min. An temperature imager was used to record the temperature changes every 5 min, as shown in Fig. 3A. The photothermal effect of AuNR@BSA and AuNR@CTAB with the same absorbance (OD = 1) is significantly higher than that of PBS. We could find that the temperature rose up rapidly in the first 5 min and then remained at about 80 °C for the rest of time, as illustrated Fig. 3A. The photothermal-induced temperature increase for PBS mostly caused by the overtone absorption of water at 1064 nm. The maximum temperature as a function of light intensity is illustrated in Fig. 3B. It was found that the photo-induced thermal temperature of AuNR@CTAB and AuNR@BSA at absorbance about 1 is proportional to light intensity. The temperature increasement is much faster for AuNR@CTAB and AuNR@BSA than that of PBS as the laser intensity getting higher. In addition, Fig. 3C shows that under the same irradiation conditions (1064 nm, 0.75 W/cm^2^), the maximum photothermal temperature is dramatically increased as the absorbance of both AuNR increasing. The photothermal properties of BSA-coated AuNR are bit better than that of CTAB coated. There is no significant change on the maximum photothermal temperature of AuNR@BSA (OD = 1, 61.1 °C) within three irradiation cycles (0.5 W/cm^2^, 10 min), which indicates the excellent photothermal stability of the prepared AuNR@BSA (Fig. 3D). Figure 3E depicts the photothermal temperature image of AuNR@CTAB (OD = 1), AuNR@BSA (OD = 1) and PBS under laser irradiation for 10 min. The maximum temperature of PBS is 44.5 °C, while the maximum temperature of the AuNR is as high as 85.5 °C. The above results demonstrated that the synthesized AuNR@BSA possesses appropriate absorption characteristics, photothermal conversion efficiency and photostability in the NIR-II range as an excellent photothermal therapeutic agent.

### In Vitro* Cytotoxicity and Photothermal Toxicity of Gold Nanorods*

**Fig. 4:**
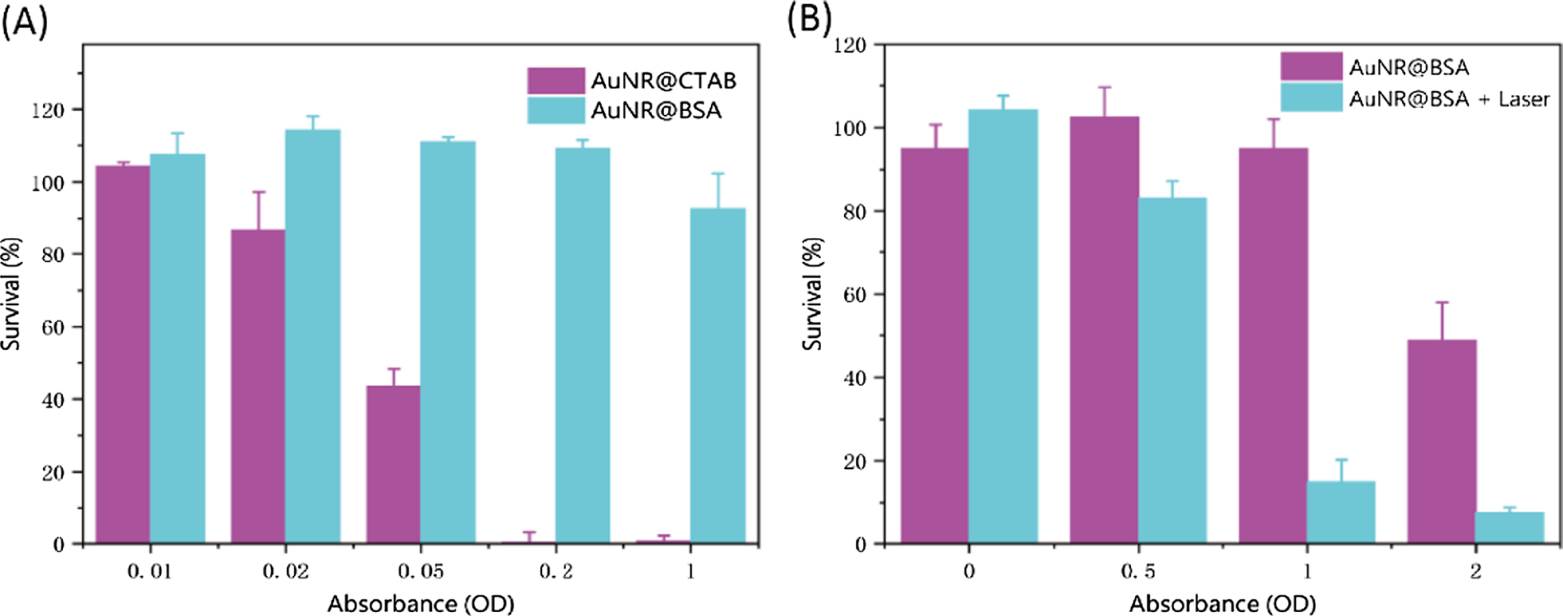
In vitro cytotoxicity and photothermal toxicity of gold nanorods. **a** Cell viability of 4T1 cells incubated with various concentrations of AuNR@CTAB and AuNR@BSA. **b** Cell viability of 4T1 cells incubated with various concentrations of AuNR@BSA without and with NIR laser irradiation (1064 nm, 0.75 W/cm^2^, 10 min). (mean ± SD, n = 3)

The CCK-8 analysis was performed to quantify the cytotoxicity of AuNR@CTAB and AuNR@BSA on 4T1 cells at different concentration. Even without laser irradiation, the AuNR@CTAB already exhibits significant cytotoxicity at very low concentration (absorbance about 0.05), hence the biological application of which is greatly restricted. However, the AuNR@BSA demonstrates excellent biological application prospects, for instance the cell viability is still in the acceptable range as the AuNR@BSA absorbance reaches to about 1 (Fig. 4A). Encouraged by the promising stability and high photothermal conversion efficiency of AuNR@BSA, the photothermal toxicity was conducted on 4T1 tumor cells in vitro with light intensity about 0.75 W/cm^2^ for 10 min. Significant photothermal toxicity was found at absorbance around 1 with cell survival rate about 20%; however, about 100% cell survivity was found for non-radiation experiments (Fig. 4B, Additional file 1: Fig. S4). These in vitro results indicate that AuNR@BSA photothermal treatment in the NIR-II region could effectively kill cancer cells at relatively radiation intensity.

### In Vivo* Biodistribution Studies*

**Fig. 5:**
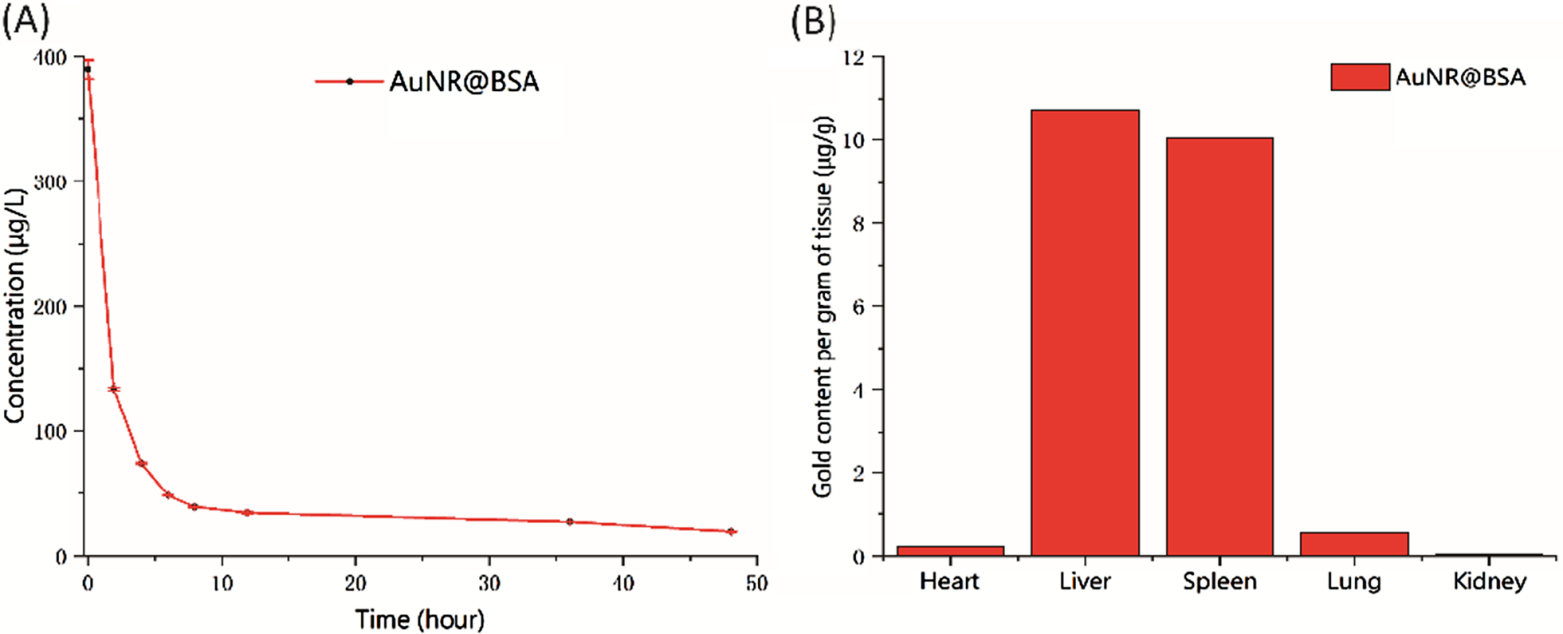
The biodistribution of AuNR@BSA in blood and organs. **a** The Au concentration in blood as a function of time via intravenous injection of AuNR@BSA. **b** The biodistribution of AuNR@BSA in various organs

Blood circulation time is essential for the successful delivery of the nanoparticle-based drugs [24]. The blood circulation time of AuNR@BSA was monitored by the Au concentration via ICP-OES and found to be about 1.5 h (half-life) (Fig. 5A). The in vivo biodistribution of AuNR@BSA was also measured by the Au concentration in different organs (Additional file 1: Fig. S5). As shown in Fig. 5B, AuNR@BSA were highly accumulated in liver and spleen after 24 h intravenous injection due to their strong phagocytosis as an organ of the reticuloendothelial system (RES) [27, 28]. These results show that AuNR@BSA can effectively accumulate in liver and spleen. AuNR@BSA has potential applications in liver and spleen-related diseases.

### In Vivo* Near-Infrared Photothermal Treatment of Gold Nanorods*

**Fig. 6:**
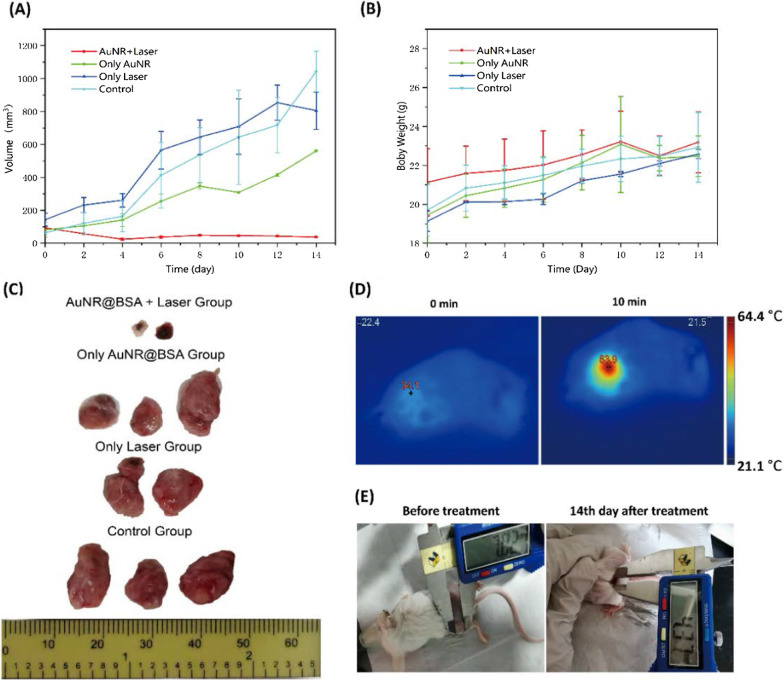
Photothermal treatment of tumor bearing mice. **a** Tumor volume as a function of time under different treatment condition. **b** Body weight changes of mice bearing tumors as a function of time, post-treatment. **c** Photographs of tumor tissues in different groups after 14-day treatment. **d** Infrared thermographic images of tumor-bearing mice exposed to a 1064 nm laser (0.75 W/cm^2^, 10 min) at 10 min post-intratumoral injection of AuNR@BSA. **e** Images of mice from AuNR@BSA_laser group after treatment. (mean ± SD, n = 2)

The excellent photothermal performance of AuNR@BSA encourage us to pursue the in vivo photothermal therapy on tumor-bearing BALB/c mice. AuNR@BSA was in situ injected into the tumor, and then, the light was introduced for photothermal therapy 10 min later (1064 nm, 0.75 W/cm^2^). The in vivo temperature variation was monitored by a thermal temperature imager. The temperature changes before and after treatment are depicted in Fig. 6D. The temperature of tumor site was about 63.9 °C within 10 min of irradiation and maintained little fluctuation at this temperature (Fig. 6D). In contrast, there is no observable temperature change for the control group in the same irradiation condition (AuNR@BSA group, PBS group, laser group). After photothermal therapy, the tumor volume and body weight of mice were monitored every two days for 14 days. As presented in Fig. 6A, B, the tumor of AuNR@BSA_laser group was completely inhibited and left as burned scabs on location of original tumor site, while that of the control groups grew relatively fast and out of control (AuNR@BSA group, PBS group, laser group). The scabs are the burned skin, which directly prove that the PPT process could cause excess local heat on the tumor site with injected AuNR@BSA. The photothermal effect is very efficient, as we observed that the solid tumors in AuNR@BSA + laser group decrease rapidly two days after treatment. To further capture the tumor changes after treatment, at the end of observation the 14-days, the mice were killed and the tumors were isolated. As shown in Fig. 6C, tumor size of the AuNR@BSA_laser treatment group was totally suppressed, while all the other groups were found to be uncontrolled growth. Images of mice from group (AuNR@BSA + laser) showed that tumors did not continue to grow after 14-day treatment (Fig. 6E). Therefore, considering the ideal therapeutic ability and no obvious cytotoxicity, AuNR@BSA for near-infrared second window laser irradiation therapy is an ideal candidate for light-triggered PTT in vivo. This clearly shows that simple hyperthermia using current NR-II therapy can effectively inhibit tumor growth (Additional file 1).

**Fig. 7:**
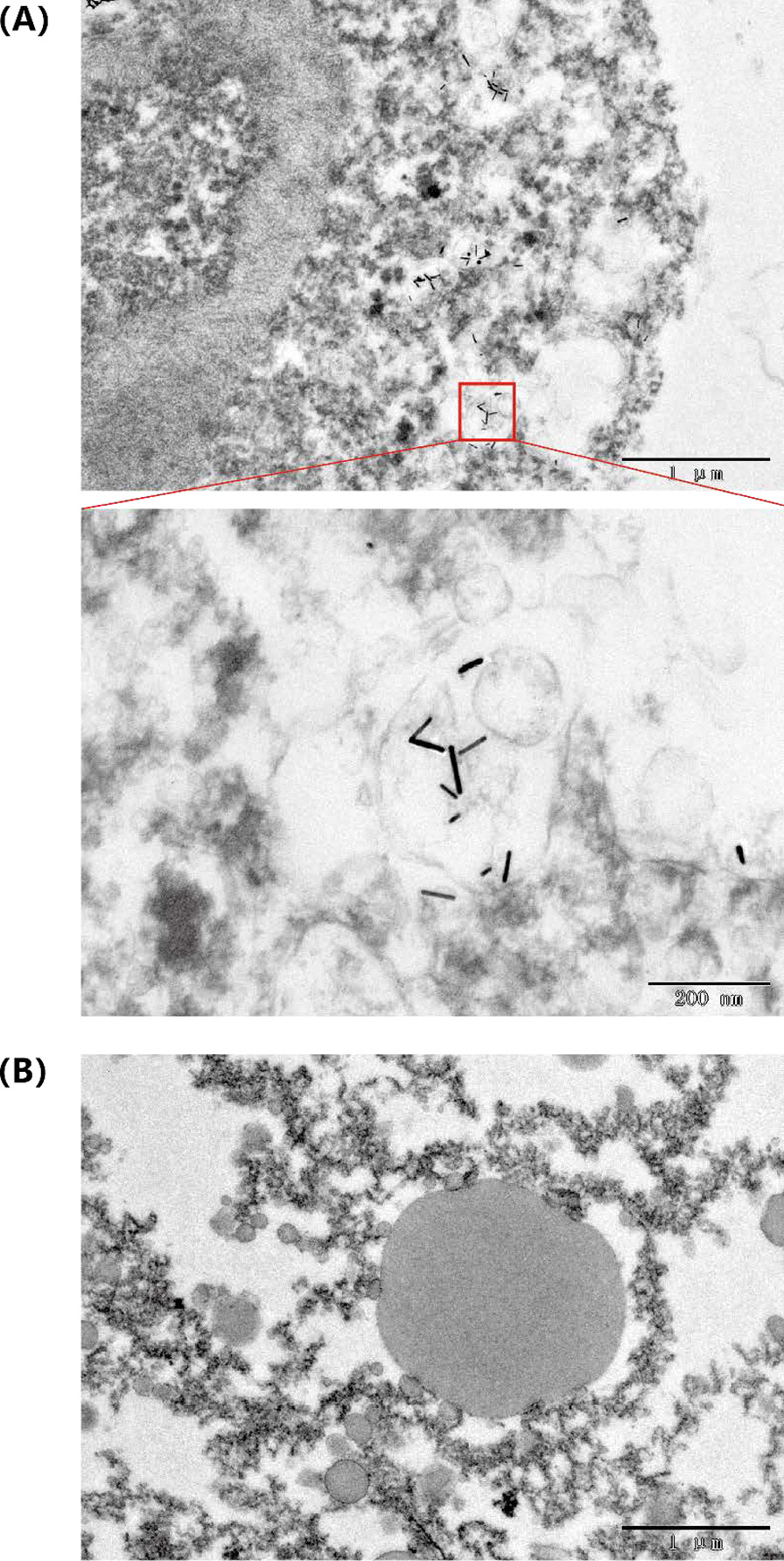
Transmission electron micrograph of AuNR@BSA in tumor tissue **a** Treated tumor. **b** Untreated tumor

In addition, TEM was taken for the isolated tumor after photothermal treatment and the untreated tumor. The AuNR could be observed in the tumor tissue after photothermal treatment. It provided further evidence that gold nanorods still have excellent photostability at tumor tissue environment (Fig. 7).

## Conclusion

Here, we synthesized the AuNR@BSA with the SPR absorption maximum in the second near-infrared window for photothermal therapy, as their outstanding photothermal properties and biocompatibility. The biocompatibility of the reported AuNR was significantly improved by coating with bovine serum albumin, and the photothermal properties were not affected. The biodistribution of the intravenously injected AuNR@BSA indicates that large amounts of AuNR accumulated in the liver and spleen. The TEM image of AuNR@BSA inside tumor reveals that the high in vivo photostability of the AuNR and suggests that once upon injection, several phototreatment might be applied to reach the desired therapy outcomes. The excellent photothermal conversion of the reported AuNR was able to sufficiently inhibit tumor growth even under low light irradiation. The PTT of AuNR@BSA combined with other treatment strategies, such as immunotherapy and chemotherapy, would be promising for developing a useful tool for personalized, safe, and effective tumor treatment.

## Supplementary Information

**Additional file 1**. Supporting information. The file contains Material characterization, List of abbreviations and Figures S1 to S5.

## Data Availability

The data set used and/or analyzed in this study can be obtained from the corresponding author upon reasonable request. All data generated or analyzed during this study is included in this published article.
