# Supplementary material for: BSA-Coated Gold Nanorods for NIR-II Photothermal Therapy
Source: Nanoscale Res Lett. 2021 Nov 29;16:170. doi: 10.1186/s11671-021-03627-7 (PMC8630206; doi:10.1186/s11671-021-03627-7)
Supplement: Supplementary file 1 — Additional file 1. Supporting information. The file contains Material characterization, List of abbreviations and Figures S1 to S5. [file 11671_2021_3627_MOESM1_ESM.docx]

**Supporting Information**

BSA-Coated Gold Nanorods for NIR-II Photothermal Therapy

Shubi Zhao^a,^ ^b, #^, Yiqun Luo^a, #^, Zong Chang^b^, Chenchen Liu^b^, Tong Li^a^, Lu Gan^a^, Yong Huang^a,^ ^⁎^, Qinchao Sun^b,^ ^⁎^

^a^ National Center for International Research of Bio-targeting Theranostics, Guangxi Key Laboratory of Bio-targeting Theranostics, Collaborative InnovationCenter for Targeting Tumor Diagnosis and Therapy, Guangxi Medical University, Nanning, Guangxi 530021, China

^b^ Research Laboratory for Biomedical Optics and Molecular Imaging, Shenzhen Institutes of Advanced Technology, Chinese Academy of Sciences, Shenzhen, Guangdong 518055, China

[Contributor Information](https://www.ncbi.nlm.nih.gov/pmc/articles/PMC6616609/#idm139697914663648aff-info).

^#^ Contributed equally.

^⁎^ Corresponding authors.

E-mail addresses: huangyong503@126.com, qchao.sun@siat.ac.cn


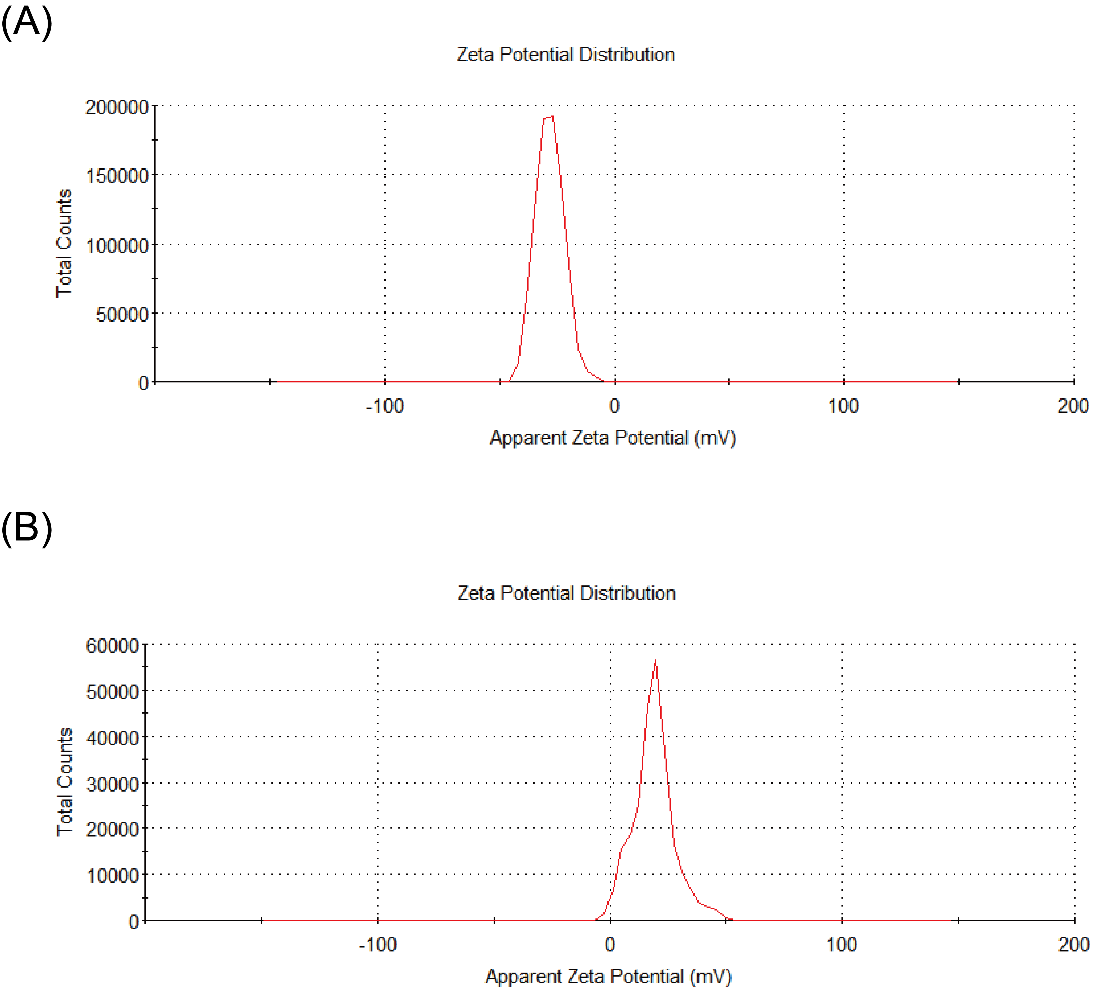


**S.1.** Characterization of AuNR@BSA and AuNR@CTAB. (A) The Zeta Potential of AuNR@BSA. (B) The Zeta Potential of AuNR@CTAB.

**
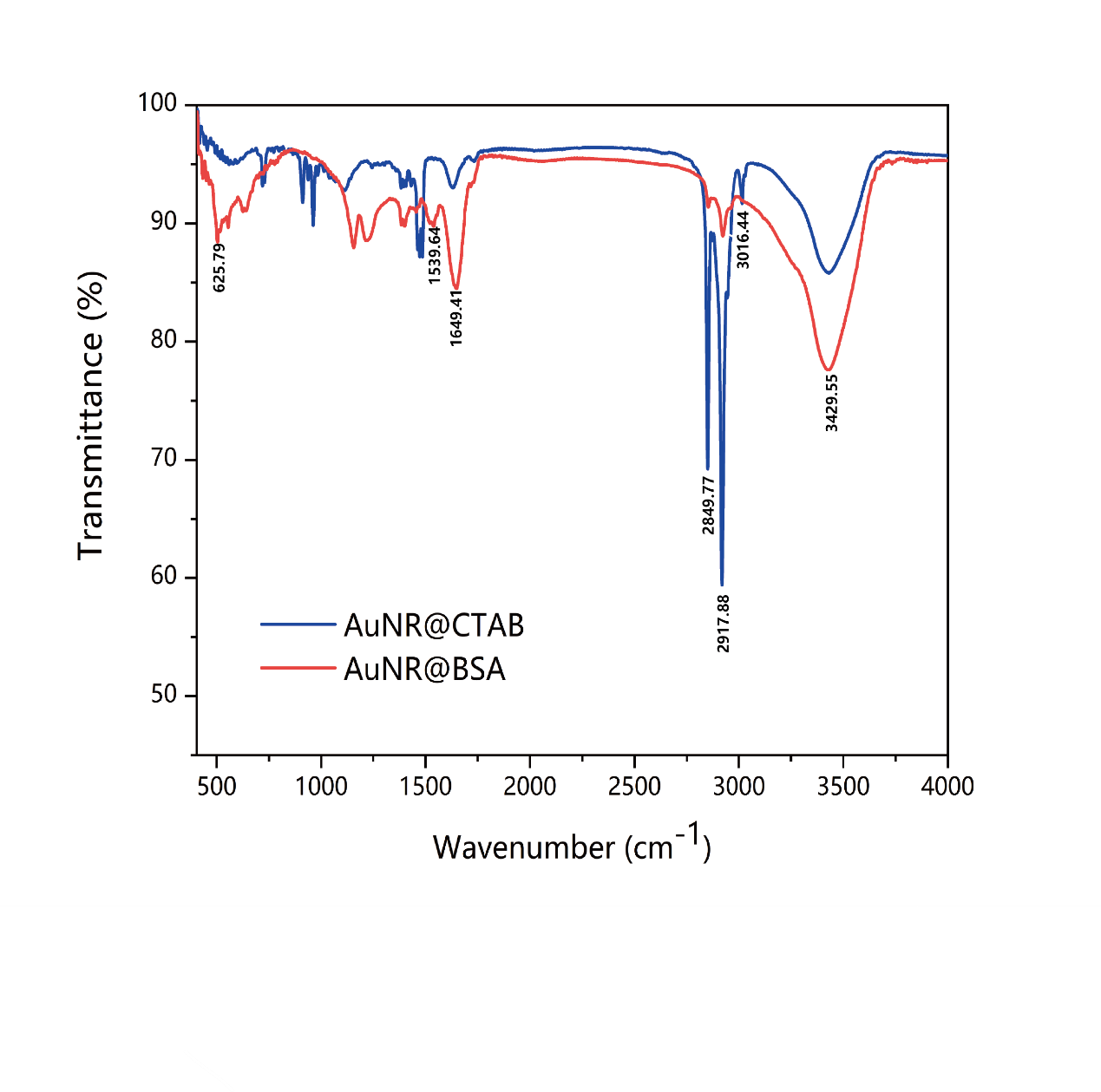
**

**S.2.** FTIR spectra of AuNR@CTAB(Blue) and AuNR@BSA(Red).


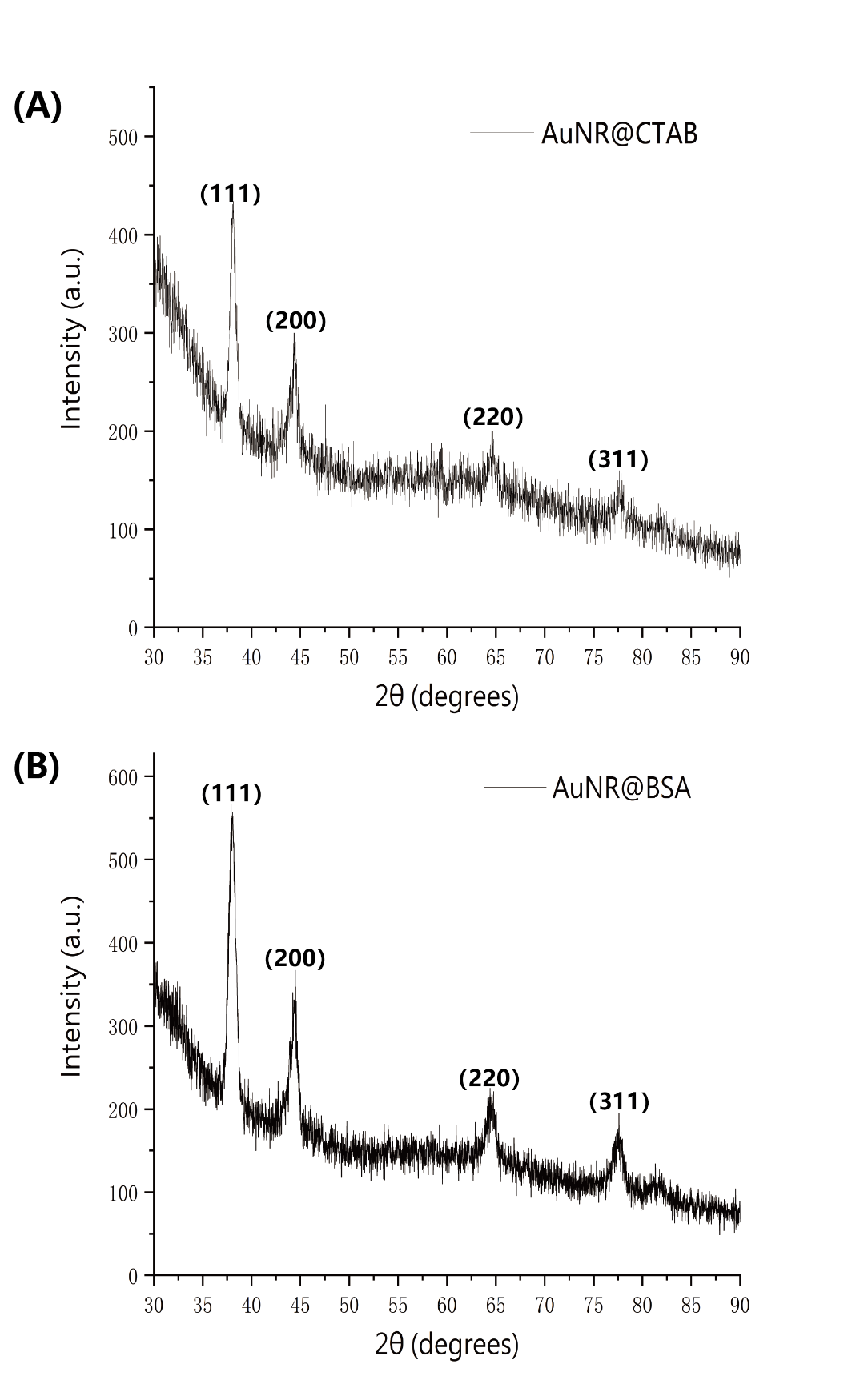


**S.3.** XRD patterns of AuNR@CTAB(A) and AuNR@BSA(B).

**
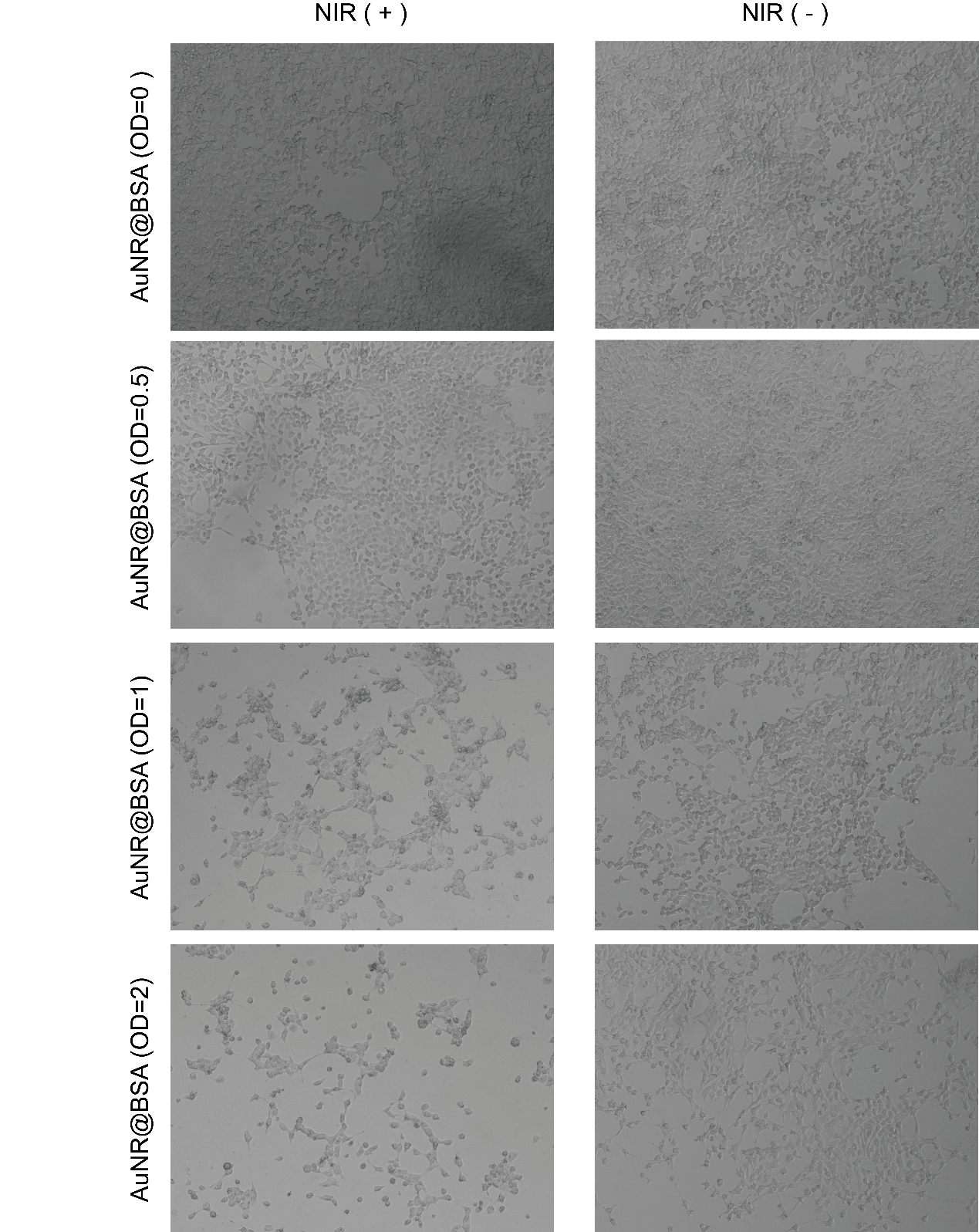
**

**S.4.** Optical image of AuNR@BSA incubated with 4T1 cells, with or without laser irradiation.


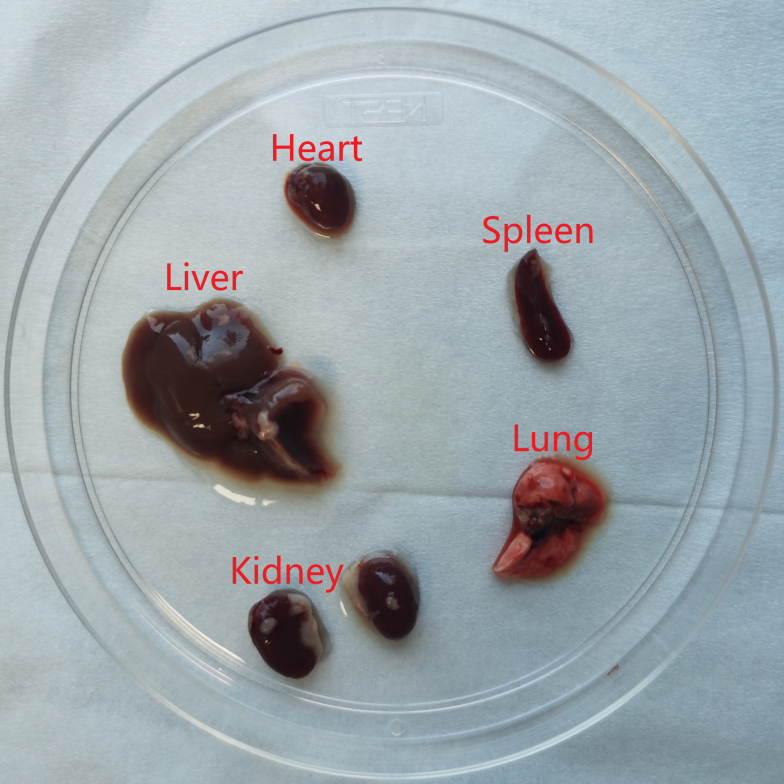


**S.5.** Optical image of the main organs of the mouse, which was injected by AuNR@BSA via the tail vein after 24 hours.

**References**

1. L. Zu, L. Liu, Y. Qin, H. Liu, H. Yang, Multifunctional BSA-Au nanostars for photoacoustic imaging and X-ray computed tomography. Nanomedicine : nanotechnology, biology, and medicine, 12(2016) 1805–1813. https://doi.org/10.1016/j.nano.2016.05.003

**List of abbreviations**

Bovine Serum Albumin (BSA), Near-infrared-II (NIR-II), Computed Tomography (CT), Surface Plasmon Resonance (SPR), Photothermal Therapy (PTT), CTAB coated gold nanorods (AuNR@CTAB), BSA coated gold nanorods (AuNR@BSA), Gold nanorods (AuNR), Transmission Electron Microscope (TEM), Dynamic Light Scattering(DLS), Gold chloride trihydrate (HAuCl_4_·3H_2_O), Hexadecyl trimethyl ammonium bromide (CTAB), Sodium borohydride (NaBH_4_), Silver nitrate (AgNO_3_), Hydrochloric acid (HCl), Guaranteed Reagent(GR), Analytical Reagent (AR), Phosphate Buffered Solution (PBS), Foetal Bovine Serum (FBS), Cell Counting Kit-8 (CCK-8), Fourier Transform Infrared spectroscopy(FT-IR), X-ray diffraction (XRD), Inductively Coupled Plasma Optical Emission Spectroscopy (ICP-OES).
